# Eltrombopag during induction chemotherapy in older patients with newly diagnosed acute myeloid leukemia is safe and reduces platelet transfusion requirements: The EPAG‐2015 French Innovative Leukemia Organization phase 2 randomized trial

**DOI:** 10.1002/cncr.70669

**Published:** 2026-10-30

**Authors:** Arnaud Pigneux, Pierre‐Yves Dumas, Marc Bernard, Norbert Vey, Audrey Bidet, Ariane Mineur, Aline Schmidt, Martin Carré, Mario Ojeda, Sarah Bertoli, Anne Banos, Pierre Peterlin, Romain Guièze, Yohan Desbrosses, Gabrielle Roth Guepin, Célestine Simand, Thibaut Leguay, Emmanuel Gyan, Eric Jourdan, Jean‐Philippe Vial, Tony Marchand, Yosr Hicheri, Marie C. Béné, Jean Francois Hamel, Christian Récher

**Affiliations:** ^1^ Hematology Clinic and Cellular Therapy Bordeaux University Hospital Pessac France; ^2^ BRIC UMR 1312 National Institute of Health and Medical Research Bordeaux University Bordeaux France; ^3^ Hematology Clinic Rennes University Hospital Rennes France; ^4^ Hematology Clinic Paoli‐Calmettes Cancer Institute Marseilles France; ^5^ Hematology Biology Bordeaux University Hospital Pessac France; ^6^ Hematology Clinic Angers University Hospital, FHU Goal Université d’Angers Université de Nantes CHU Angers National Institute of Health and Medical Research, CNRS, CRCI2NA, SFR ICAT Angers France; ^7^ Hematology Clinic Grenoble Alpes University Hospital La Tronche France; ^8^ Hematology Clinic Mulhouse Hospital Mulhouse France; ^9^ Institut Universitaire du Cancer de Toulouse Oncopole Toulouse University Hospital Toulouse University Toulouse France; ^10^ Hematology Clinic Bayonne Hospital Bayonne France; ^11^ Hematology Clinic Nantes University Hospital Nantes France; ^12^ Hematology Clinic Clermont Ferrand University Hospital Clermont Ferrand France; ^13^ Hematology Clinic Besançon University Hospital Besançon France; ^14^ Hematology Clinic Nancy University Hospital Nancy France; ^15^ Hematology Clinic Strasbourg University Hospital Strasbourg France; ^16^ Hematology Clinic and Cellular Therapy Tours University Hospital Tours France; ^17^ Hematology Clinic Nîmes University Hospital Nîmes France; ^18^ Hematology Biology Nantes University Nantes France; ^19^ Clinical Research Center Angers University Hospital Angers France

**Keywords:** acute myeloid leukemia, elderly patients, eltrombopag, induction chemotherapy, platelet transfusion, toxicity

## Abstract

**Background:**

Elderly patients with acute myeloid leukemia (AML) treated with intensive chemotherapy are at risk of developing thrombopenia, which requires platelet transfusions. It would be interesting to provide them with thrombopoietin receptor agonists such as eltrombopag.

**Methods:**

The EPAG‐2015 multicenter phase 2 trial (18 French hospitals from October 2018 to June 2021) blindly randomized elderly patients (aged ≥60 years) with placebo or eltrombopag administered on Day 11 after induction chemotherapy and continued until AML response evaluation (Day 45) or a platelet count of >100 × 10^9^/L. A cohort of 110 patients with a median age of 70 years was included and randomized (55 per arm) to receive placebo or eltrombopag.

**Results:**

After induction, 90 patients (81.8%) achieved complete response (CR) or CR with incomplete reconstitution (CRi) (45 in each group), including 74 patients with CR (38 with eltrombopag and 36 with placebo). With a median follow‐up for alive patients of 20.9 months the primary objective was not reached, given that there was no difference in overall survival (hazard ratio, 1.59; 95% CI, 0.65–3.89; *p* = .31). Eltrombopag, however, did not lead to increased grade 3/4 adverse events, and was associated with a lower median number of platelet transfusions (eight; interquartile range [IQR], 6–14; vs. 10; IQR, 7–14; i.e., a 20% benefit).

**Conclusions:**

This study failed to demonstrate a survival advantage after eltrombopag addition, at variance with previous reports, but shows that initiating eltrombopag after chemotherapy washout is feasible and safe, and resulted in a decreased need for platelet transfusions.

## Abstract 2

In the EPAG‐2015 double‐blind, phase 2 randomized trial, addition of eltrombopag at Day 11 of induction, up to platelet reconstitution or Day 45, did not improve outcomes compared to the placebo group. However, addition of this thrombopoietin agonist allowed a decrease in the need for platelet transfusion, without increased toxicity.

## INTRODUCTION

Acute myeloid leukemia (AML) is the most common form of acute leukemia in adults. The median age of patients at diagnosis is 68 years, and elderly subjects indeed present a higher risk of developing AML (20.1 per 100,000 after age 65 years).^1^, ^2^, ^3^, ^4^ The prognosis for elderly patients with AML is particularly poor, given a lower tolerance to intensive chemotherapies, comorbidities, poor‐risk cytogenetics, and/or multidrug resistance. Consequently, the overall survival of these patients rarely exceeds 10% beyond 5 years from diagnosis. For this group of patients, improving the efficacy of induction chemotherapy and preventing relapse, without increasing treatment‐related morbidity and mortality, remain a challenge.^2^

In patients with AML, blast proliferation in the bone marrow (BM) progressively leads to BM failure that translates into peripheral cytopenias. Chemotherapy itself aggravates these low blood cell counts, and hematologic toxicities are the rule during treatment. Grade 4 thrombocytopenia, nearly unavoidable in the treatment of AML, can lead to life‐threatening hemorrhage. Classically, this is avoided by platelet transfusions; however, these are not always well tolerated, and platelet half‐life is relatively short after transfusion (1–5 days).^5^ Patients can also become refractory to platelet transfusions, which thereby reduces their therapeutic potential.^6^ Other ways of preventing thrombocytopenia should therefore be investigated to improve the management of patients with AML, especially elderly ones.

Eltrombopag is a small molecule, orally bioavailable, and an agonist of the thrombopoietin receptor, that stimulates thrombopoiesis.^7^ It also has been shown to act on both AML cells, by limiting their proliferation,^8^ and normal hematopoietic precursor cells, by stimulating them, and thus potentially contributing to hematologic reconstitution.^9^ Two phase 1 studies in small numbers of patients, published as abstracts, have suggested a possible benefit in controlling the disease of de novo^10^ or refractory/relapsed^11^ patients with AML. This led to the initiation of the design of the EPAG‐2015 trial. However, later publications, although confirming increased platelet counts, did not demonstrate better outcomes, and even reported rather high toxicity.^12^, ^13^, ^14^, ^15^

For the French Innovative Leukemia Organization (FILO) group, the standard of care for induction chemotherapy for elderly fit patients with AML includes lomustine as a third drug added to standard‐dose cytarabine and anthracycline.^16^ Adding eltrombopag to this chemotherapy regimen, and thus reducing at the same time the leukemic burden and rate of fatal hemorrhage while decreasing platelet transfusion needs, seemed to be an attractive option to improve outcomes in elderly patients treated for AML. Results of the EPAG‐2015 trial, which explored this option, are presented here.

## MATERIALS AND METHODS

EPAG‐2015 was designed as a randomized, double‐blind versus placebo, phase 2 multicenter trial aiming at assessing the impact of eltrombopag administered to elderly patients with AML receiving induction chemotherapy (NCT03603795). The trial was conducted in 18 centers of the FILO group in France, mostly university hospitals. It had been approved by the French Health Authority on October 12, 2015 (150913A‐11), and by the Comité de Protection des Personnes Sud‐Ouest and Outre Mer on December 12, 2015 (2015/117). The trial was conducted according to the principles of the Declaration of Helsinki and International Conference on Harmonization Good Clinical Practice Guidelines. All patients provided written informed consent. Patient sex/gender data were collected according to their individual social security number, which corresponds to the biological sex assessed at birth.

### Procedures

Randomization was performed by the FILO data manager for EPAG‐2015, with the sponsor, pharmacies, investigators, and patients blinded to the arm allotted. Randomization used a preestablished table and a 1:1 ratio. Inclusion criteria were as follows: age of ≥60 years, newly diagnosed AML (de novo or therapy‐related, with the exception of acute promyelocytic leukemia and acute megakaryoblastic leukemia), favorable or intermediate cytogenetic risk according to the Medical Research Council (MRC) 2010 classification (available at trial design),^17^ Eastern Cooperative Oncology Group performance status of <3, Hematopoietic Cell Transplantation Comorbidity Index (HCT‐CI) score of <3, and adequate baseline organ function.

Exclusion criteria were AML with adverse cytogenetic risk or arising from myelodysplastic syndrome (MDS), myeloproliferative neoplasm/chronic myelomonocytic leukemia, or *BCR::ABL1* translocation, known active central nervous system (CNS) history of a thromboembolic event, or ongoing use of anticoagulation.

The induction chemotherapy regimen (Figure S1) consisted of one cycle of daunorubicin 60 mg/m^2^/day, Days 1–3, cytarabine 100 mg/m^2^/day, Days 1–7, and lomustine 200 mg/m^2^, orally on Day 1. Daunorubicin was chosen over idarubicin because of its faster pharmacokinetics,^18^ and because it is less likely to interfere with eltrombopag and therefore provides a clearer washout. Eltrombopag 200 mg (provided by Novartis Pharmaceuticals) or placebo (blinded similar tablets) was given orally once daily from Day 11 until platelet counts reached 100 × 10^9^/L or until AML response evaluation (maximum Day 45). Per recommendation, oral administration was instructed to be performed on an empty stomach. The decision regarding platelet transfusion was left to each physician; however, the 2015 recommendation of the French Health Authority has a 10 × 10^9^/L threshold. The use of granulocyte colony‐stimulating factor, an antifungal, antiviral, and anti–*Pneumocystis jirovecii* prophylaxis, was recommended. Patients who failed to reach complete response (CR) or CR with incomplete hematologic recovery (CRi)^4^, ^19^ after this single cycle of induction were off study, whereas patients with CR or CRi were postulated to receive up to six courses of outpatient consolidation every 30–45 days with daunorubicin 60 mg/m^2^, Day 1, and cytarabine 50 mg/m^2^/12 h, subcutaneously, Days 1–5. Patients then received 6 months of maintenance therapy alternating mercaptopurine and methotrexate.^16^ The addition of midostaurin for patients with *FLT3*‐ITD or *FLT3*‐TKD mutations was not allowed, given the potential interactions mentioned in eltrombopag specificities. Allogeneic hematopoietic stem cell transplantation (Allo‐HSCT) was recommended after two to four cycles in patients with intermediate or adverse risk according to the European LeukemiaNet (ELN) 2017 classification, which had been published at that time.^19^

Patients were evaluated on Day 11, before eltrombopag initiation, and on Day 45, after induction, for clinical, biological, and toxicity assessment. Then, treatment, biological, and toxicity parameters were collected after each consolidation.

The primary objective^20^ of the study was to improve overall survival (OS). Secondary objectives were (1) CR and CRi rates, disease‐free survival (DFS), and event‐free survival (EFS) at 12 months; (2) OS at 2, 3, and 5 years; (3) days with a platelet count of <10 × 10^9^/L; (4) time to a platelet count of >100 × 10^9^/L; (5) percentage of patients with a platelet count of >100 × 10^9^/L at Day 45; (6) number of platelet transfusions, with an objective of a 10% reduction in the eltrombopag group compared to placebo; (7) time to a hemoglobin of >8 g/dL; (8) number of red blood cell transfusions; (9) time to an absolute neutrophil count of >0.5 × 10^9^/L; and (10) safety assessment of eltrombopag via National Cancer Institute Common Terminology Criteria for Adverse Events, version 4.03.

### Molecular analysis

Genomic DNA from BM was extracted by standard procedures and centrally sequenced with an Illumina NextSeq 550Dx sequencer and the Magnis SureSelect XT HS capture panel (Agilent, Santa Clara, California) covering the complete coding regions (and −2 to +2 splicing sites) of 58 genes recurrently mutated in myeloid neoplasms, as described in Supporting Information S1.

### Statistical analysis

The primary analysis compared survival rates depending on the treatment arm (eltrombopag vs. placebo). The actual 12‐month survival rate for patients not treated with eltrombopag was 50% when the trial was designed.^21^ A 12‐month survival rate increase of approximately 20% was postulated for patients treated with eltrombopag. A type I error set at 10%, a type II error set at 20%, and an expected survival rate of 50% in the control arm, which highlighted a 20% improvement in survival rate via a two‐tailed log‐rank test, required 110 patients (55 patients in the eltrombopag arm and 55 in the control arm). A type I error set at 10% was used for all other parameters, especially for the reduction of platelet transfusions.

Data monitoring was performed by clinical research assistants via the monitoring tool of the electronic case report form.

Continuous data are reported as medians and interquartile ranges (IQRs), and were compared via a two‐tailed Student *t*‐test or nonparametric Mann–Whitney *U* test as appropriate. Categorical data are reported as numbers and percentages, and were compared via a two‐tailed χ^2^ or Fisher exact test when appropriate.

The occurrence of events over time and according to treatment arm was studied via Kaplan–Meier curves and one‐tailed log‐rank tests.

Response rates, recovery of 100 × 10^9^/L platelet count, and severe thrombotic events were studied with a 45‐day follow‐up. The overall response rate comprised patients in CR/CRi.

OS, DFS, and EFS were ultimately evaluated at 12‐ and 36‐month follow‐up (there were not enough patients at 5 years), calculated respectively from treatment initiation until death or last date of follow‐up, from CR/CRi until relapse, death, or last date of follow‐up, or from treatment initiation to failure, relapse, death, or last day of follow‐up.

Analyses were performed with Stata 13.1 (College Station, Texas) and MedCalc (Ostend, Belgium) statistical software.

## RESULTS

From October 2018 until June 2021, 110 patients (71 males; 64.5%) with a median age of 70 years (IQR, 66–73 years) were included. As shown in Figure S2, they were randomized to receive standard therapy, supplemented by placebo or eltrombopag (55 patients in each arm). Patient characteristics at enrollment are shown in Table 1. Patients in both arms were largely comparable; however, those randomized to receive eltrombopag had significantly fewer previous histories of cancer (three vs. 12; *p* = .01). Notably, although they remained in the intention‐to‐treat analysis, five patients were wrongly included: one in the eltrombopag arm (HCT‐CI, >2) and four in the placebo arm (one with an adverse karyotype, two with an HCT‐CI score of >2, and one with inadequate cardiac function). Thus, the whole population of 110 patients was included in the analysis. Most patients had intermediate‐risk cytogenetics (97.3%) according to MRC criteria^17^ (one failure and three with core‐binding factor translocations) (Table S1). Classification according to ELN 2022^4^ criteria (*n* = 100 patients with retrospective molecular analyses) indicated that 22 patients could be classified as favorable risk, 16 as intermediate risk, and the majority (*n* = 62) as high risk. The 10 patients without molecular data had an intermediate karyotype.

**TABLE 1:** Patient characteristics.

Characteristic	All (*N* = 110)	Eltrombopag (*n* = 55)	Placebo (*n* = 55)	*p*
Sex				
Male, No. (%)	71 (64.55)	32 (58.18)	39 (70.91)	NS
Age, median (IQR), years	70 (66–73)	70 (67–73)	70 (65–74)	NS
History of cancer, No. (%)	15 (13.76)	3 (5.45)	12 (22.22)	.01
ECOG performance status, No. (%)				
0	43 (39.45)	24 (44.44)	19 (34.55)	NS
1	60 (55.05)	29 (53.70)	31 (56.36)	NS
2	6 (5.50)	1 (1.85)	5 (9.09)	NS
BMI > 25, No. (%), kg/m^2^	73 (66.36)	34 (61.82)	39 (70.91)	NS
Hemoglobin, median (IQR), g/dL	9.25 (8.3–10.8)	9.3 (8.4–11.3)	9.10 (8.2–10.4)	NS
Platelets, median (IQR), 10^9^/L	62.5 (31–109)	65 (32–85)	61 (27–105)	NS
Leukocytes, median (IQR), 10^9^/L	2.73 (1.6–8.7)	3.32 (1.8–8.6)	2.5 (1.52–8.9)	NS
ANC, median (IQR), 10^9^/L	0.7 (0.27–1.86)	0.8 (0.24–1.89)	0.57 (0.28–1.4)	NS
Peripheral blasts, median (IQR), 10^9^/L	0.21 (0.00–3.42)	0.28 (0.01–3.62)	0.09 (0.6–2.82)	NS
BM blasts, median (IQR), %	40.5 (25–65)	39 (26–63)	41 (22–73)	NS
HCT‐CI (Sorror), median (IQR)	0 (0–2)	0 (0–2)	0 (0–2.7)	NS
Intermediate Kyt, No. (%)	106 (97.2)	53 (98.1)	53 (96.3)	NS
2022 ELN risk classification, No.	100	49	51	
Favorable, No. (%)	22 (22)	13 (26.5)	9 17.6)	NS
Intermediate, No. (%)	16 (16)	8 (16.3)	8 (15.7)	
Adverse, No. (%)	62 (62)	28 (57.4)	34 (66.7)	
Mutations, No.	100	49	51	
*NPM1*, No. (%)	24 (24)	14 (28.6)	10 (19.6)	NS
*FLT3*‐ITD, No. (%)	6 (6)	3 (6.1)	3 (5.9)	NS
*FLT3*‐TKD, No. (%)	10 (10)	6 (12.2)	4 (7.8)	NS
*CEBPA*, No. (%)	12 (12)	7 (14.2)	5 (9.8)	NS
*DNMT3A*, No. (%)	18 (18)	6 (12.2)	12 (23.5)	NS
*IDH1*, No. (%)	10 (10)	6 (12.2)	4 (7.8)	NS
*IDH2*, No. (%)	14 (14)	7 (14.2)	7 (13.7)	NS
*RUNX1*, No. (%)	29 (29)	12 (24.5)	17 (33.3)	NS
*ASXL1*, No. (%)	29 (29)	11 (22.4)	18 (35.3)	NS
*TP53*, No. (%)	4 (4)	1 (2.0)	3 (5.9)	NS

Abbreviations: ANC, absolute neutrophil count; BM, bone marrow; BMI, body mass index; ECOG, Eastern Cooperative Oncology Group; ELN, European LeukemiaNet; HCT‐CI, Hematopoietic Cell Transplantation Comorbidity Index; IQR, interquartile range; Kyt, karyotype; NS, not significant.

On Day 11, when eltrombopag or placebo was initiated, median platelet counts were 21 × 10^9^/L (IQR, 10–31 × 10^9^/L) in the eltrombopag arm and 24 × 10^9^/L (IQR, 13–34 × 10^9^/L) in the placebo group.

Two patients in the eltrombopag group did not receive the drug: one because of severe nausea and swallowing difficulties, and the other by patient choice. The daily treatment was four tablets per day, as per protocol. The median duration of eltrombopag treatment was 19 days (IQR, 14–21 days). Treatment was interrupted before Day 45 for 31 of 53 treated patients, mostly (*n* = 18) because the platelet count had already reached 100 × 10^9^/L. Other causes were failure or progression (*n* = 3), patient choice (*n* = 1), and omitted intake of 1 day (*n* = 1). For the eight other patients, the reason was toxicity, as detailed in Table S1.

In the placebo group, four patients did not receive the placebo tablets (septic thrombophlebitis, *n* = 1; thrombosis, *n* = 1; exclusion criteria, *n* = 2). Placebo was interrupted in 33 of 51 patients, also mostly because platelets had reached 100 × 10^9^/L (*n* = 22). The other reasons were physician decision for two patients, and patient choice for two. For the last seven patients, the reason was toxicity (*n* = 2), as shown in Table S1, failure or progression (*n* = 2), mesenteric ischemia (*n* = 2) and CR evidenced on BM aspiration (*n* = 1).

At the time of evaluation, the median follow‐up time for alive patients was 20.9 months (95% CI, 19.11–23.96 months). The primary objective of improved OS was not reached. Median OS was 25.8 months (95% CI, 18.13 months to not evaluable [NE]) and 25.7 months (95% CI, 20.07 months to NE), respectively, for patients in the eltrombopag or placebo arm (Figure 1). There was no significant difference in the 12‐month OS of 78.0% (95% CI, 64.5%–86.9%) and 85.3% (95% CI, 72.8%–92.4%) between the eltrombopag and placebo groups, respectively. Median DFS was, respectively, 17.81 months (95% CI, 12.98 months to NE) and 23.52 months (95% CI, 14.59–39.29 months) (Figure 1). Moreover, 12‐month DFS was, respectively, 66.7% (95% CI, 50.9%–78.4%) and 73.0% (95% CI, 57.4%–83.7%). Median EFS was 12.9 months (95% CI, 7.13–18.13 months) and 13.14 months (95% CI, 2.79–20.6 months) in the eltrombopag and placebo arms, respectively (Figure 1), whereas 12‐month EFS was 52.7% (95% CI, 38.8%–64.9%) versus 50.5% (95% CI, 36.6%–62.8%), respectively, all without a statistically significant difference.

**FIGURE 1:**
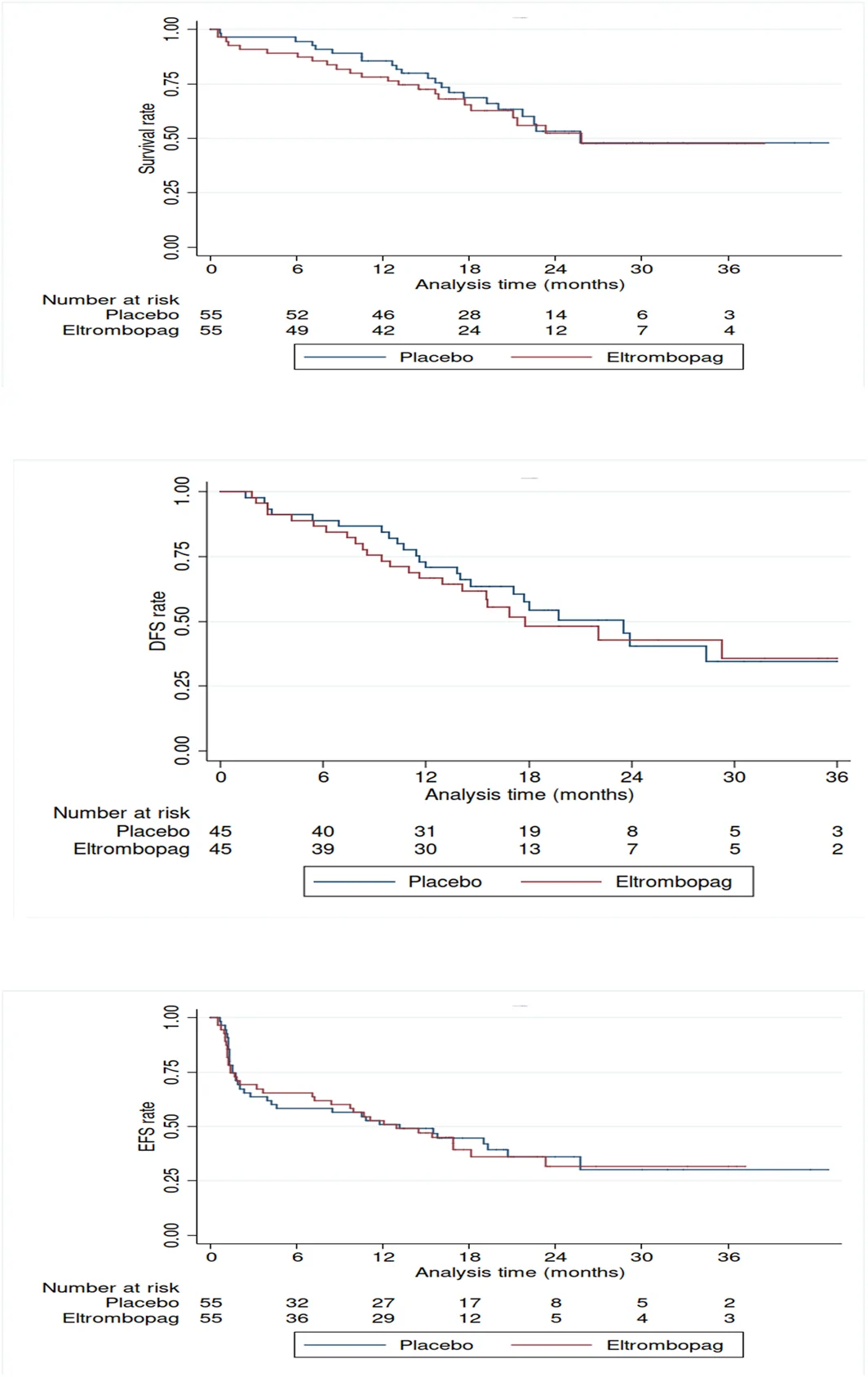
Kaplan–Meier curves for overall, disease‐free, and event‐free survival. DFS indicates disease‐free survival; EFS, event‐free survival.

Three‐year OS remained not statistically significantly different, with 41.0% (95% CI, 25.7%–55.8%) of patients alive in the eltrombopag group versus 43.0% (95% CI, 25.7%–55.8%) in the placebo group. Three‐year DFS was, respectively, 38.2% (95% CI, 20.6%–55.6%) and 41.2% (95% CI, 22.4%–59.1%). Three‐year EFS was identical, at 23.6% (95% CI, 12.5%–36.7%).

No difference was observed in OS, DFS, or EFS over the first year of treatment between risk groups according to ELN 2022^4^ that could be applied via retrospective molecular analyses (Figure S3).

After induction (Figure S2), 90 patients (81.8%) achieved CR/CRi (45 in each group) after the single induction cycle, including 74 patients with CR (38 [69%] in the eltrombopag group and 36 [65%] in the placebo group). Respectively, seven and nine patients reached CRi, whereas three (one and two, respectively) obtained a partial response, 11 failed (five and six, respectively), and six died early (four and two, respectively).

Table 2 indicates the parameters assessed at the postinduction evaluation (maximum Day 45, at a median of 37 days; IQR, 34–41 days), which showed no difference between the two arms. Overall, the median time to a platelet recovery of >100 × 10^9^/L was 30 days (IQR, 27–34 days), without any difference between the two arms, although this delay was slightly shorter in the eltrombopag arm. Median platelet counts at Day 45 were 258 × 10^9^/L (IQR, 94–969 × 10^9^/L) in the eltrombopag group and 228 × 10^9^/L (IQR, 96–364 × 10^9^/L) in the placebo group.

**TABLE 2:** Outcome at evaluation.

Parameter	Eltrombopag	Placebo	*p*
Time with platelet count < 10 × 10^9^/L, median (IQR), days	2 (0–13)	4 (0–14)	.73
Time to platelet count ≥ 100 × 10^9^/L, median (IQR), days	30 (2733)	31 (27–35)	.34
Platelet count > 100 × 10^9^/L at Day 45, No. (%)	34 (61.8)	38 (69)	.42
Platelet transfusions, median (IQR)	8 (6–14)	10 (7–14)	.064
Time to hemoglobin ≥ 8 g/dL, median (IQR), days	22 (18–26)	22 (17–26)	.84
RBC transfusions, median (IQR)	8 (6–10)	9 (6–12)	.44
Time to ANC ≥ 0.5 × 10^9^/L, median (IQR), days	23 (17–26)	23 (19–26)	.17

Abbreviations: ANC, absolute neutrophil count; IQR, interquartile range; RBC, red blood cell.

The median number of platelet transfusions was significantly reduced in the eltrombopag group, at eight (IQR, 6–14) versus 10 (IQR, 7–14) in the placebo group (*p* = .06; significant at the assumed 10% [0.1] risk), namely a 20% benefit. More significantly, post hoc analysis showed that 11 patients in the placebo arm required 20 platelet transfusions or more during induction, whereas this was the case for only two in the eltrombopag arm (*p* = .008). There was no difference in the number of red blood cell transfusions.

Of the 90 responding patients, 85 entered the consolidation phase. Two patients in the eltrombopag group did not receive the consolidation program: one because of Allo‐HSCT performed after induction failure, and one for cardiac comorbidity.

Twenty‐eight patients (25.5%) received Allo‐HSCT in the first CR: 13 (23.6%) after eltrombopag, and 15 (27.3%) after placebo.

Toxicities during induction (Table 3) were comparable in both groups, except for a median of 2 more days of fever in patients who received eltrombopag. Notably, a similar rate of grade 3/4 general toxicity was observed in both arms (eltrombopag, *n* = 22 patients [40%], vs. placebo, *n* = 26 patients [47%]; *p* = .44). Severe (grade 3/4) hemorrhages occurred in one patient who received eltrombopag versus three who received placebo (not significant). CNS toxicity was observed only in two placebo patients. Thrombotic events were recorded in one patient who received eltrombopag (not considered attributable to the drug) and two who received placebo.

**TABLE 3:** Adverse events during induction chemotherapy.

Parameter	All (*N* = 110)	Eltrombopag (*n* = 55)	Placebo (*n* = 55)	*p*
Hemorrhage grade 3/4, No.	4	1^a^	3^b^	NS
Duration with fever ≥ 38°C, median (IQR), days	7 (3–11)	7 (3–12)	5 (3–9)	.06
Duration of iv antibiotics, median (IQR), days	20 (14–27)	19 (14–25)	21 (15–28)	.20
Patients with grade 3 or 4 general toxicity,^c^ No.	48	22	26	.44
Cutaneous	7	4	3	NS
Mucosal	5	2	3	NS
Diarrhea	5	3	2	NS
Vomiting	1	1	0	NS
Constipation	1	1^d^	0	
Nausea	0	0	0	
Pulmonary (infections)	8	5	3	NS
Cardiac	7	5^e^	2^f^	NS
Central neurologic	2	0	1^g^	NS
Hepatic	11	5^h^	6^i^	NS
Renal	0	0	0	
Thrombosis	3	2^j^	1^k^	
Mesenteric ischemia	2	1	1	
Infection^l^	12	3	9	NS

*Note:* See also Table S1.

Abbreviations: IQR, interquartile range; iv, intravenous.

^a^ Fatal hemorrhagic shock Day 3 eltrombopag.

^b^ Gingivorrhagia grade 3, resolved; extradural hematoma grade 3, resolved; and one fatal stroke ischemic and secondary hemorrhagic, grade 5.

^c^ Some patients had several events.

^d^ Occlusive syndrome, ileus grade 3.

^e^ Hypertension grade 3, resolved; acute myocardial infarction at Day 16, grade 3, resolved; alteration in left ventricular ejection fraction, grade 3, resolved; and acute coronary syndrome in two (Days 3 and 11) resolved.

^f^ Hypertension.

^g^ Multifactorial coma, grade 3, resolved.

^h^ Increase in liver enzymes in five patients, and cholestasis in one patient.

^i^ Increase in liver enzymes.

^j^ Portal vein thrombosis Day 3, grade 3, resolved, and superficial vein thrombosis Day 22, grade 3, resolved.

^k^ Superficial vein thrombosis, grade 3, resolved.

^l^ Two fatal infections in the eltrombopag arm, and one in the placebo arm.

## DISCUSSION

The EPAG‐2015 trial’s primary objective of improving 1‐year OS by adding eltrombopag to induction chemotherapy was not reached. However, the addition of eltrombopag succeeded in reducing platelet transfusions without increased toxicity.

The rationale of the trial was to test the antileukemic action of this drug and its ability to decrease the complications of thrombocytopenia in intensive chemotherapy. The results do not provide any signal of a better antitumoral effect, despite the expectations based on the in vitro and clinical data available at the time of study design.^7^, ^8^ Although there is no clear explanation for this failure, it can be noted that these studies were very preliminary. Besides, early clinical studies used eltrombopag as monotherapy^10^, ^11^, ^12^ and sometimes continuously for a longer time^14^ than in the EPAG‐2015 trial. Moreover, with the availability of alternative therapies such as azacitidine, or the azacitidine–venetoclax combination for the frailest patients, those selected to receive intensive therapy are fitter and also benefit from the current safer management, with de facto improved outcomes. For instance, here, because a single severe hemorrhage occurred in the placebo group and none in the eltrombopag arm, this could not have been improved.

It can also be hypothesized that the 200‐mg dosage was not optimal. On the basis of the 200‐ to 300‐mg efficacy range reported previously,^22^ the lowest dose was chosen to limit toxicities. Indeed, Frey et al.^14^ commented on possibly dose‐related excess mortality and severe hemorrhages. Whether the duration of exposure could have been prolonged can also be argued. However, treatment could be suspended if platelet counts reached 100 × 10^9^/L, which occurred for a rather large number of patients before Day 45, although eltrombopag was initiated at Day 11 of induction. Another reason to avoid higher dosages was the historical concern of eltrombopag stimulating leukemic blast growth, which has been refuted by Roth et al.^8^ and was not observed here.

Finally, 1‐year OS rates in both arms of EPAG‐2015 (85% and 78% in the eltrombopag arm and placebo group, respectively), without any difference for either at 3 years, are higher than expected, which likely reflects the improvement in short‐term outcomes observed in most studies of patients with AML.^23^

The EPAG‐2015 trial was focused on other possible positive effects of the addition of eltrombopag after Day 11, notably with the aim of reducing platelet transfusions by 10%. In fact, the ultimate reduction of platelet transfusions was by 20%, which suggests the efficacy of eltrombopag in maintaining platelet levels. Globally, platelet recovery was better in the eltrombopag arm, with higher platelet counts than in the placebo arm at evaluation. In the study by Frey et al.^14^ there was no reduction in platelet transfusions during induction, which was explained by the authors by the myelosuppressive effects of chemotherapy countering the effect of eltrombopag. This was also the case in the ASPIRE trial,^22^ where eltrombopag was used as monotherapy for severely thrombopenic patients with intermediate‐2 or high‐risk MDS or AML in active disease, where a reduction in clinically relevant thrombocytopenic events was obtained, although without yielding transfusion independence. In EPAG‐2015, it can be argued that initiating eltrombopag earlier and at a higher dosage could have led to a more marked reduction in platelet transfusions. Yet, as mentioned above, a conservative strategy was used with the major aim of reducing toxicity, which was achieved.

Indeed, another positive result of the EPAG‐2015 trial is that toxicity was globally low, with no excess toxicity in the eltrombopag arm. Notably, a similar rate of grade 3/4 adverse events was observed in both arms, with no significant difference in terms of either hemorrhagic or thrombotic events or deaths. The trial reported by Frey et al.,^14^ which had a design comparable to that of EPAG‐2015 and combined intensive chemotherapy and eltrombopag in a multicenter randomized study, differed in that it did not show any efficacy and was associated with great toxicity. The differences are that this international study enrolled a much younger population and included 22% of patients with poor cytogenetics (not included in EPAG‐2015). At variance with EPAG‐2015, a high rate of adverse events occurred in this trial, with 9% (vs. 1%) of grade 3–5 bleeding and an alarming death rate of 53% (vs. 41%) in the eltrombopag arm. Another explanation for these differences is that in the study by Frey et al.,^14^ eltrombopag was given until platelet counts reached 200 × 10^9^/L, patients were in CR, or up to 42 days. Moreover, only 4 days separated daunorubicin and eltrombopag initiation, which was approximately 20 h after the end of Day 3 daunorubicin infusion. At that time, daunorubicin, which has a half‐life of 26.7 h, was not yet eliminated. In the EPAG‐2015 trial, daunorubicin was chosen and administered from Day 1 to 3 in order to avoid interaction with eltrombopag pharmacokinetics. Indeed, this led the FILO group to change to daunorubicin instead of the usual idarubicin^16^ because the idarubicin active metabolite, idarubicinol, has an elimination half‐time of 55 h.^18^ The absence of severe hemorrhage could thus be due to the delayed introduction of eltrombopag after elimination of anthracycline metabolites, as had been suggested by Frey et al.^14^ These authors actually indicated as a conclusion of their work that a delayed introduction of eltrombopag could prove more efficient and less toxic, which is probably the reason for the safest results of the EPAG‐2015 trial. More recently, results of the phase 2 DELTA trial^24^ were published, where eltrombopag or placebo was added sequentially (Days 12–25 of 28‐day cycles) to a hypomethylating agent (azacitidine or decitabine) for elderly patients with AML ineligible to receive intensive chemotherapy. Although there was no added toxicity (as in the EPAG‐2015 trial), no benefit was observed compared to the placebo group. It must be noted, however, that these patients were older, had a poorer prognosis, and were more thrombocytopenic than in the present report.

The advantages and limitations of the EPAG‐2015 trial have been described above. Another limitation could be that it is lacking power, given the rather small size of the cohorts evaluated.

In conclusion, although the results of the EPAG‐2015 trial did not demonstrate an improvement in survival, they suggest that the addition of eltrombopag after the elimination of daunorubicin decreases the need for platelet transfusions after induction chemotherapy, and could be prescribed safely in treatment schedules using this anthracycline.

## AUTHOR CONTRIBUTIONS

**Arnaud Pigneux:** Conceptualization, investigation, funding acquisition; writing–original draft, validation, writing–review and editing, supervision, and formal analysis. **Pierre‐Yves Dumas:** Investigation. **Marc Bernard:** Investigation. **Norbert Vey:** Investigation. **Audrey Bidet:** Investigation. **Ariane Mineur:** Investigation and project administration. **Aline Schmidt:** Investigation. **Martin Carr** **é** **:** Investigation. **Mario Ojeda:** Investigation. **Sarah Bertoli:** Investigation. **Anne Banos:** Investigation. **Pierre Peterlin:** Investigation. **Romain Gui** **è** **ze:** Investigation. **Yohan Desbrosses:** Investigation. **Gabrielle Roth Guepin:** Investigation. **C** **é** **lestine Simand:** Investigation. **Thibaut Leguay:** Investigation. **Emmanuel Gyan:** Investigation. **Eric Jourdan:** Investigation. **Jean‐Philippe Vial:** Investigation. **Tony Marchand:** Investigation. **Yosr Hicheri:** Investigation. **Marie C. B** **é** **n** **é** **:** Investigation, writing–original draft, methodology, and formal analysis. **Jean Francois Hamel:** Investigation, formal analysis, methodology, and writing–review and editing. **Christian R** **é** **cher:** Methodology and investigation.

## CONFLICT OF INTEREST STATEMENT

Sarah Bertoli reports consulting for Servier Affaires Medicales, AbbVie, Astellas Pharma, and Celgene. Pierre Peterlin reports consulting for Servier Pharmaceuticals, and receiving travel support from Servier Pharmaceuticals, AbbVie, and Daiichi Sankyo. Romain Gui è ze reports receiving grants from Johnson & Johnson, Lilly, Roche, BeiGene, AstraZeneca, and AbbVie, and consulting for Johnson & Johnson. Emmanuel Gyan reports consulting for Gilead Sciences, Novartis, and Astellas Pharma. Christian R é cher reports receiving grants from Astellas Pharma, Daiichi Sankyo, Bristol‐Myers Squibb, AbbVie Biotherapeutics, and IQVIA, and consulting for Daiichi Sankyo, Bristol‐Myers Squibb, Astellas Pharma, Novartis Pharma, Janssen Global Services, AbbVie Biotherapeutics, and Servier Affaires Medicales. The other authors declare no conflicts of interest.

## Supporting information

Supplementary Material

## Data Availability

The data that support the findings of this study are available from the corresponding author on reasonable request.

## References

[1] Short NJ , Rytting ME , Cortes JE . Acute myeloid leukaemia. Lancet. 2018;392(1047):593‐606. doi:10.1016/s0140-6736(18)31041-9 PMC10230947 30078459

[2] Estey EH . Acute myeloid leukemia: 2021 update on risk‐stratification and management. Am J Hematol. 2020;95(11):1368‐1398. doi:10.1002/ajh.25975 32833263

[3] Abdallah M , Xie Z , Ready A , Manogna D , Mendler JH , Loh KP . Management of acute myeloid leukemia (AML) in older patients. Curr Oncol Rep. 2020;22(10):103. doi:10.1007/s11912-020-00964-1 PMC8026174 32725515

[4] Döhner H , Wei AH , Appelbaum FR , et al. Diagnosis and management of AML in adults: 2022 recommendations from an international expert panel on behalf of the ELN. Blood. 2022;140(12):1345‐1377. doi:10.1182/blood.2022016867 35797463

[5] Leader A , Hofstetter L , Spectre G . Challenges and advances in managing thrombocytopenic cancer patients. J Clin Med. 2021;10(6):1169. doi:10.3390/jcm10061169 PMC8000983 33799591

[6] Kantarjian H , Giles F , List A , et al. The incidence and impact of thrombocytopenia in myelodysplastic syndromes. Cancer. 2007;109(9):1705‐1714. doi:10.1002/cncr.22602 17366593

[7] Kuter DJ . The biology of thrombopoietin and thrombopoietin receptor agonists. Int J Hematol. 2013;98(1):10‐23. doi:10.1007/s12185-013-1382-0 23821332

[8] Roth M , Will B , Simkin G , et al. Eltrombopag inhibits the proliferation of leukemia cells via reduction of intracellular iron and induction of differentiation. Blood. 2012;120(2):386‐394. doi:10.1182/blood-2011-12-399667 PMC3398759 22627766

[9] Kao YR , Chen J , Narayanagari SR , et al. Thrombopoietin receptor‐independent stimulation of hematopoietic stem cells by eltrombopag. Sci Transl Med. 2018;10(458):eaas9563. doi:10.1126/scitranslmed.aas9563 PMC9899005 30209246

[10] Mittelman M , Assouline S , Briasoulis E , et al. Eltrombopag treatment of thrombocytopenia in advanced myelodysplastic syndromes and acute myeloid leukemia: results of the 8‐week open‐label part of an ongoing study. Blood. 2012;120(21):3822. doi:10.1182/blood.v120.21.3822.3822

[11] Frey NV , Hexner EO , Porter DL , et al. A phase I clinical trial using eltrombopag in patients with acute myelogenous leukemia. Blood. 2012;120(21):3576. doi:10.1182/blood.v120.21.3576.3576

[12] Platzbecker U , Wong RS , Verma A , et al. Safety and tolerability of eltrombopag versus placebo for treatment of thrombocytopenia in patients with advanced myelodysplastic syndromes or acute myeloid leukaemia: a multicentre, randomised, placebo‐controlled, double‐blind, phase 1/2 trial. Lancet Haematol. 2015;2(10):e417‐e426. doi:10.1016/s2352-3026(15)00149-0 26686043

[13] Mittelman M , Platzbecker U , Afanasyev B , et al. Eltrombopag for advanced myelodysplastic syndromes or acute myeloid leukaemia and severe thrombocytopenia (ASPIRE): a randomised, placebo‐controlled, phase 2 trial. Lancet Haematol. 2018;5(1):e34‐e43. doi:10.1016/s2352-3026(17)30228-4 29241762

[14] Frey N , Jang JH , Szer J , et al. Eltrombopag treatment during induction chemotherapy for acute myeloid leukaemia: a randomised, double‐blind, phase 2 study. Lancet Haematol. 2019;6(3):e122‐e131. doi:10.1016/s2352-3026(18)30231-x 30704923

[15] Mukherjee S , Li H , Hobbs BP , et al. A single arm, phase II study of eltrombopag to enhance platelet count recovery in older patients with acute myeloid leukemia (AML) undergoing remission induction therapy. Blood. 2019;134(1):2595. doi:10.1182/blood-2019-132178

[16] Pigneux A , Harousseau JL , Witz F , et al. Addition of lomustine to idarubicin and cytarabine improves the outcome of elderly patients with de novo acute myeloid leukemia: a report from the GOELAMS. J Clin Oncol. 2010;28(18):3028‐3034. doi:10.1200/jco.2009.26.4648 20479424

[17] Grimwade D , Hills RK , Moorman AV , et al. Refinement of cytogenetic classification in acute myeloid leukemia: determination of prognostic significance of rare recurring chromosomal abnormalities among 5876 younger adult patients treated in the United Kingdom Medical Research Council trials. Blood. 2010;116(3):354‐365. doi:10.1182/blood-2009-11-254441 20385793

[18] Robert J . Clinical pharmacokinetics of idarubicin. Clin Pharmacokinet. 1993;24(4):275‐288. doi:10.2165/00003088-199324040-00002 8491056

[19] Döhner H , Estey E , Grimwade D , et al. Diagnosis and management of AML in adults: 2017 ELN recommendations from an international expert panel. Blood. 2017;129(4):424‐447. doi:10.1182/blood-2016-08-733196 PMC5291965 27895058

[20] Cheson BD , Bennett JM , Kopecky KJ , et al. Revised recommendations of the International Working Group for Diagnosis, Standardization of Response Criteria, Treatment Outcomes, and Reporting Standards for Therapeutic Trials in Acute Myeloid Leukemia. J Clin Oncol. 2003;21(24):4642‐4649. doi:10.1200/jco.2003.04.036 14673054

[21] Pigneux A , Béné MC , Guardiola P , et al. Addition of androgens improves survival in elderly patients with acute myeloid leukemia: a GOELAMS study. J Clin Oncol. 2017;35(4):387‐393. doi:10.1200/jco.2016.67.6213 28129526

[22] Mittelman M , Platzbecker U , Grosicki S , Lawniczek T , Zhu Z , Selleslag D . Long‐term safety and efficacy of eltrombopag for advanced myelodysplastic syndromes or acute myeloid leukemia and severe thrombocytopenia: results of the ASPIRE extension study. Acta Haematol. 2023;146(5):373‐378. doi:10.1159/000531146 PMC10614231 37231838

[23] Othus M , Kantarjian H , Petersdorf S , et al. Declining rates of treatment‐related mortality in patients with newly diagnosed AML given 'intense' induction regimens: a report from SWOG and MD Anderson. Leukemia. 2014;28(2):289‐292. doi:10.1038/leu.2013.176 PMC4457325 23760400

[24] Sockel K , Röllig C , Mütherig A , et al. Hypomethylating therapy with or without eltrombopag in elderly patients with acute myeloid leukemia: results from the randomized, placebo‐controlled phase 2 DELTA trial. Am J Hematol. 2025;100(12):2436‐2441. doi:10.1002/ajh.70091 41036595

