# Supplementary material for: Eltrombopag during induction chemotherapy in older patients with newly diagnosed acute myeloid leukemia is safe and reduces platelet transfusion requirements: The EPAG‐2015 French Innovative Leukemia Organization phase 2 randomized trial
Source: Cancer. 2026 Oct 30;132(21):e70669. doi: 10.1002/cncr.70669 (PMC13722882; doi:10.1002/cncr.70669)
Supplement: Supplementary file 1 [file CNCR-132-e70669-s001.docx]

**Eltrombopag or placebo during induction chemotherapy in older patients with newly diagnosed acute myeloid leukemia. The EPAG-2015 FILO phase II randomized trial.**

Arnaud Pigneux, MD, PhD,^1,2^ Pierre-Yves Dumas, MD, PhD,^2^ Marc Bernard, MD,^3^ Norbert Vey, MD,^4^ Audrey Bidet, PharmD,^5^ Ariane Mineur,^1^ Aline Schmidt, MD, PhD,^6^ Martin Carré, MD,^7^ Mario Ojeda, MD,^8^ Sarah Bertoli, MD, PhD,^9^ Anne Banos, MD,^10^ Pierre Peterlin, MD,^11^ Romain Guièze, MD, PhD,^12^ Yohan Desbrosses, MD,^13^ Gabrielle Roth Guepin, MD,^14^ Célestine Simand, MD,^15^ Thibaut Leguay, MD,^1^ Emmanuel Gyan, MD, PhD,^16^Eric Jourdan, MD,^17^ Jean-Philippe Vial, MD,^5^ Tony Marchand, MD, PhD,^3^ Yosr Hicheri, MD,^4^ Marie C Béné, PharmSciD, PhD,^18^ Jean Francois Hamel, MD,^19^ and Christian Récher, MD, PhD.^9^

**Supplemental material**

Supplementary Figure S1. Trial schedule.

Supplementary Figure S2. Flowchart

*Maintenance alternating mercaptopurine and methotrexate except for 4 patients (azacitidine n=3, midostaurine and eltrombopag n=1)

**Supplementary Figure S3. 12 month survival curves according to ELN 2022 classification.** For the favorable, intermediate and adverse groups, median OS were respectively not evaluable (NE), NE and 23.36 (95%CI 16.59-NE) months, DFS were 29.30 (95%CI 11.40-NE), 23.88 (95%CI 16.5.45-NE) and 17.81 (95%CI 13.80-23.52) months and EFS (10.84 (95%CI 1.28-NE), 18.13 (95%CI 3.68-NE) and 12.09 (95%CI 3.25-19.03) months. There was no statistically significant difference between the three groups for the three outcomes,

**Supplemental Table S1.** Detail of toxicities that led to interruption of the drug tested.

|  | **Eltrombopag N= 55** | **Placebo N= 55** | |
| --- | --- | --- | --- |
| Hemorrhage | - Aspiration pneumonia and hemorrhagic shock at day 3, grade 3 then 5, fatal |  |  |
| Constipation | - Occlusive syndrome (ileus) at day 12, grade 3, resolved, treatment resumed |  |  |
| Nausea | - Persisting nausea on day 7, grade 2, resolved |  |  |
| Cardiac | - Acute myocardial infarct at day 16, grade 3, resolved - Acute coronary syndrome at day 11, grade 2, resolved |  |  |
| Hepatic | - Cholestatic liver injury at day 27, grade 2, resolved | - Liver enzymes increase at day 17, grade 3, resolved - Liver enzymes increase at day 21, grade 3, resolved |  |
| Thrombosis | - Portal vein thrombosis at day 3, grade 3, resolved | - Ischemic stroke of the left anterior artery, with secondary hemorrhagic changes, at day 16, grade 4 then 5, fatal |  |
| Infection | - Neutropenic colitis with mesenteric ischemia at day 4, grade 3 then 5, fatal | - Septic shock with mesenteric ischemia at day 13, fatal |  |

**Supplemental material. Molecular analyses.**

Raw NGS data were analyzed using different variant callers:

- Mutect2, https://gatk.broadinstitute.org/hc/en-us/profiles/388875408552-GATK-Team
- Varscan2, https://github.com/Jeltje/varscan2
- Lofreq, https://csb5.github.io/lofreq/
- Vardicts, https://github.com/serbod/NovaLib/blob/master/VarDicts.pas

Variant interpretation was performed considering minor allele frequencies (MAF) in the public GnomAD (https://gnomad.broadinstitute.org/) database of polymorphisms (variants with MAF >0.02 in overall population/global ancestry or sub-continental ancestry are excluded), variant allele frequencies (VAF), prevalence and clinical interpretation (COSMIC, protein impact [https://cancer.sanger.ac.uk/cosmic]).

All variants were checked manually on IGV (https://bioinformatics-core-shared-training.github.io/intro-to-IGV/InspectingVariantsInIGV.html) and named according to the Human Genome Variation Society (https://www.hgvs.org/).

**List of tested genes**

*ANKRD26, ASXL1, ASXL2, BCOR, BCORL1, CALR, CBL, CCND2, CEBPA, CSF3R, CUX1, DDX41, DHX15, DNMT3A, ETNK1, ETV6, EZH2, FLT3, GATA1, GATA2, GNAS, GNB1, IDH1, IDH2, IKZF1, JAK2, KDM6A, KIT, KRAS, MPL, MYC, NFE2, NPM1, NRAS, PHF6, PPM1D, PTEN, PTPN11, RAD21, RIT1, RUNX1, SAMD9, SAMD9L, SETBP1, SF3B1, SH2B3, SMC1A, SMC3, SRSF2, STAG2, TET2, TERC, TERT, TP53, U2AF1, UBA1, WT1 and ZRSR2.*
